# Thermal Annuloplasty for the Treatment of Discogenic Low Back Pain With a High-Intensity Zone After Full Endoscopic Discectomy

**DOI:** 10.7759/cureus.73795

**Published:** 2024-11-16

**Authors:** Tatsuya Maegawa, Kotaro Kohara, Eitaro Okumura, Ryo Hashimoto, Motoo Kubota

**Affiliations:** 1 Spinal Surgery, Kameda Medical Center, Chiba, JPN; 2 Neurosurgery, Tokyo Women's Medical University, Tokyo, JPN

**Keywords:** full endoscopic discectomy, full endoscopic spinal surgery, high intensity zone, low back pain, thermal annuloplasty

## Abstract

Discogenic low back pain (DLBP) is difficult to diagnose. We performed full endoscopic spinal surgery (FESS) with thermal annuloplasty for DLBP and achieved good results. Here, we report a case in which thermal annuloplasty resulted in good outcomes for refractory DLBP accompanied by a residual high-intensity zone (HIZ) after full endoscopic discectomy (FED).

The patient was a 22-year-old female with low back pain (LBP) that worsened on bending forward. Magnetic resonance imaging (MRI) revealed bulging at the L4/L5 level and lumbar disc herniation (LDH) at the L5/S1 level. The condition worsened but then improved over time. However, since she began working as a nurse a year prior to presentation, her symptoms worsened again, and she has experienced severe LBP and left sciatica. Since MRI showed a slight increase in the L5/S1 LDH, FED (interlaminar approach at the left L5/S1 level) was performed, and her symptoms in the left lower limb quickly disappeared. The LBP also improved, but when she returned to work, the pain worsened. Oral medications had little effect; therefore, she underwent periodic block injections. MRI revealed that the LDH at the L5/S1 level had disappeared, but a small HIZ lesion remained. LBP worsened on discography and improved with a disc block. The condition was diagnosed as DLBP accompanied by HIZ. Thermal annuloplasty was performed, resulting in the immediate disappearance of the LBP.

An HIZ indicates inflammation; cauterization can suppress this inflammation and improve discogenic pain. The histopathological findings included angiogenesis and inflammatory cell infiltration. DLBP accompanied by an HIZ may develop after FED (transforaminal approach), and thermal annuloplasty is as effective as the usual HIZ. To our knowledge, this is the first report of thermal annuloplasty for the treatment of postoperative HIZ.

## Introduction

Low back pain (LBP) includes many types of pain (nociceptive, neuropathic, nociplastic, and nonspecific) that frequently overlap. Due to the many contributing factors associated with LBP and the low specificity of imaging and diagnostic injections, the best method for diagnosing this condition remains controversial [1].

Treatment depends primarily on pain classification and usually begins with self-care and pharmacotherapy combined with non-pharmacological methods such as physiotherapy and psychotherapy. For refractory LBP, carefully selected patients are offered a wide range of nonsurgical (e.g., epidural steroid injections for neuropathic pain or radiofrequency ablation and intra-articular steroid injections for mechanical pain) and surgical (e.g., decompression for neuropathic pain or disc replacement and fusion for mechanical causes) treatment options [2]. Additionally, the use of full endoscopic spinal surgery (FESS) for the treatment of LBP has also been reported [3].

FESS is now widely used as a surgical treatment for lumbar disc herniation (LDH), and full endoscopic discectomy (FED) is one of the least invasive surgical procedures of this type. In a meta-analysis comparing FED, microendoscopic discectomy (MED), and open discectomy (OD), the postoperative visual analog scale (VAS) and Oswestry Disability Index indicated comparably good outcomes, and there was no difference in the incidence of complications. However, the operative time of FED was significantly shorter than that of OD (54.6 vs. 102.6 minutes, p=0.0001), and the estimated blood loss tended to be lower during FED than during OD (3.3 vs. 244.9 mL, p=0.07), demonstrating the safety and usefulness of FED [4].

Discogenic low back pain (DLBP) is one of the various forms of LBP and refers to all degenerative diseases of the lumbar intervertebral discs that do not have nerve tissue compression as the main manifestation [2,5]. It has multiple and complex causes and is thus difficult to diagnose. However, the introduction of the concept that DLBP is defined as LBP due to disruption of the nucleus pulposus and the destruction of the annulus fibrosus without apparent herniation has resulted in a deep understanding of LBP and its importance for clinical diagnosis and treatment [6]. Discography is currently recognized as the gold standard for the diagnosis of DLBP. However, the pathogenesis of DLBP has not been completely clarified, and no specific diagnostic methods have been established yet.

High-intensity zones (HIZs) on magnetic resonance imaging (MRI) of the lumbar spine were first reported in 1992 by Aprill and Bogduk and refer to a localized high signal area located at the posterior end of the annulus fibrosus, away from the nucleus pulposus, on T2-weighted images of the lumbar spine [7]. Aprill and Bogduk demonstrated that HIZs can cause pain in approximately 90% of cases when contrast media leaks due to annulus fibrosus rupture during discography, and this is known as pain replication. Therefore, it has been suggested that HIZs are a major sign of DLBP. However, subsequent studies have shown contrasting results, showing that HIZs are also present in 24% of asymptomatic individuals and that 40% of discographies without HIZs were positive [8]. Nevertheless, some meta-analyses with similar content were published to evaluate the correlation between HIZs and the gold standard discography in the diagnosis of DLBP [9]. These evidence-based meta-analyses highlighted the mechanism of correlation between HIZs and a positive lumbar discography.

There have been reports of FESS with thermal annuloplasty for patients with an HIZ [10]. Similarly, we performed thermal annuloplasty under these conditions and achieved good results. We report a case in which thermal annuloplasty was performed for refractory DLBP accompanied by a residual HIZ after FED, and a good outcome was obtained. To our knowledge, there have been no other reports of surgical treatment for postoperative HIZ.

## Case presentation

**Figure 1:**
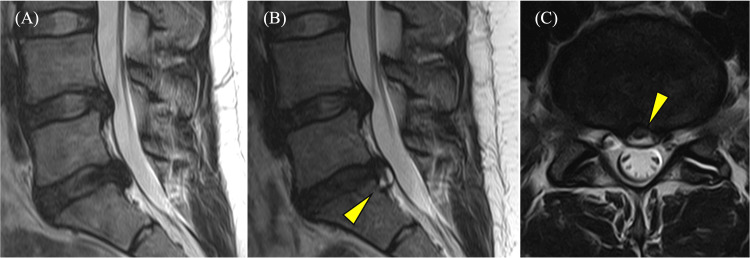
MRI before the first surgery. Sagittal T2-weighted MRI (A) three years prior showing bulging or lumbar disc herniation at the L4/L5 and L5/S1 levels. Sagittal (B) and axial (C) T2-weighted MRI taken before the first surgery showing no change in bulging at the L4/L5 level, worsening compression of the dural sac, and appearance of a high-intensity zone (arrowhead) at the L5/S1 level. MRI, magnetic resonance imaging

A 22-year-old female working as a nurse had been experiencing LBP for three years. MRI at that time showed bulging at the L4/L5 level and lumbar disc herniation (LDH) at the L5/S1 level (Figure 1A). She had been visiting the hospital for conservative treatments such as pharmacological therapy. Since then, her symptoms have repeatedly worsened and remitted. She has sometimes been aware of numbness in the left S1 region. LDH at the L5/S1 level was the primary cause of symptoms. Since she started working as a nurse one year ago, her symptoms have worsened. She experienced LBP, sciatica, and numbness in the left lower limb. MRI showed a slight increase in LDH at the L5/S1 level with a clear and widespread HIZ (Figures 1B, 1C).

**Figure 2:**
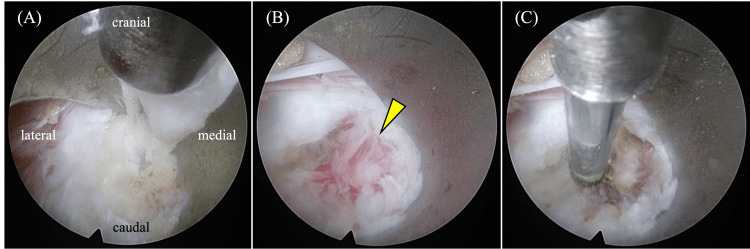
First surgery (full endoscopic discectomy) Image taken during the first surgery (full endoscopic discectomy, interlaminar approach) from the left side at the L5/S1 level. (A) Removing lumbar disc herniation using a micro-rongeur. (B) Intradiscal granulation tissue with bleeding (arrowhead). (C) Thermal coagulating at the end of the surgery.

An FED-interlaminar approach (FED-ILA) was performed from the left side at L5/S1, and the symptoms in the left lower limb resolved quickly (Figure 2). LBP also improved but relapsed upon returning to work. Oral medication and physical therapy during rehabilitation were not effective, while caudal epidural block injections were temporarily effective. Therefore, the patient was administered regular caudal epidural block injections. However, because continuing work and conservative treatment resulted in severe mental and physical discomfort, it was necessary to consider other treatment options.

**Figure 3:**
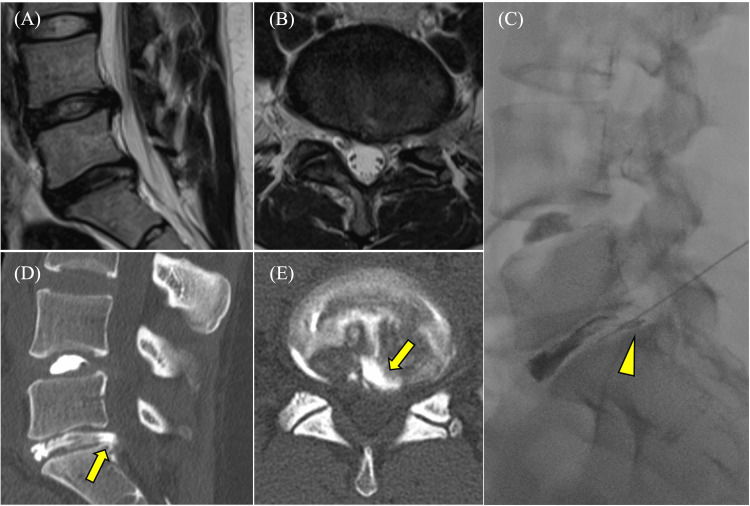
Magnetic resonance imaging, discography, and computed tomography myelography before the second surgery. Sagittal (A) and axial (B) T2-weighted magnetic resonance imaging taken before the second surgery showing near disappearance of the lumbar disc herniation but continued presence of a small high-intensity zone on the dorsal side at the L5/S1 disc. Discography (C) at L4/L5 showing normal findings and at L5/S1 showing a suspected annular tear (arrowhead); this was followed by disc block injection at the L5/S1 level. Sagittal (D) and axial (E) computed tomography myelography showing the same findings as on discography and pooling of the contrast medium inside the dorsal annulus (arrow).

Nine months after FED-ILA, MRI showed no significant changes in bulging at the L4/L5 level, and the LDH at the L5/S1 level had almost disappeared; however, a small HIZ remained on the dorsal side at the L5/S1 disc (Figures 3A, 3B). Nevertheless, as the cause of the LBP was not clear from MRI findings alone, discography was performed, which revealed normal findings at the L4/L5 level and annular tears at the L5/S1 level. Computed tomography myelography showed the same findings as discography and pooling of contrast media inside the dorsal annulus (Figures 3C-3E). Increased internal pressure at the L4/L5 disc did not induce much LBP but that at the L5/S1 disc clearly induced LBP. Moreover, disc block injection at L5/S1 improved the LBP. Finally, we diagnosed refractory DLBP accompanied by a residual HIZ after FED.

**Figure 4:**
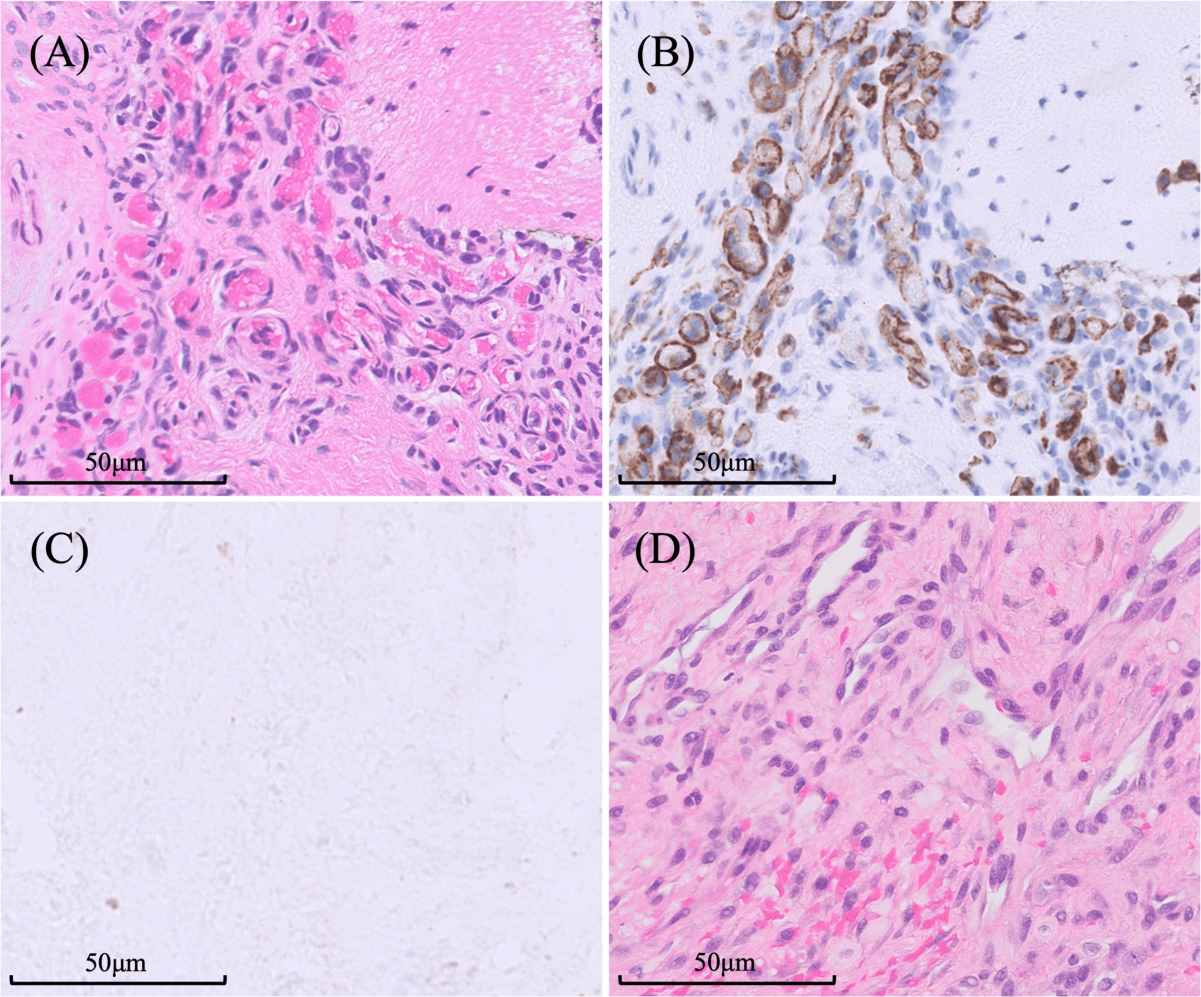
Histopathological findings. Histopathological slide showing granulation tissue rich in angiogenesis seen in herniated disc material removed during the first surgery. (A) Hematoxylin and eosin stain showing infiltration of the inflammatory cells, including macrophages, lymphocytes, and a small number of neutrophils. Immunohistochemistry showing (B) CD34 positivity and (C) S100 protein negativity. (D) Hematoxylin and eosin stain of granulation tissue specimen removed during the second surgery showing findings similar to those of the first surgery (A-D; magnification 200x).

Subsequently, thermal annuloplasty using the posterolateral approach (TA-PLA) was performed from the left side at L5/S1, and the LBP immediately resolved. The details of the surgical procedure will be described later. Histopathological findings included granulation tissue rich in angiogenesis with CD34 positivity and inflammatory cell infiltration (macrophages, lymphocytes, and a small number of neutrophils). No nerve fibers were detected, and the S-100 protein test was negative. In addition, the histopathological findings were exactly the same in the first and second surgeries (Figure 4).

Thermal annuloplasty (posterolateral approach)

**Figure 5:**
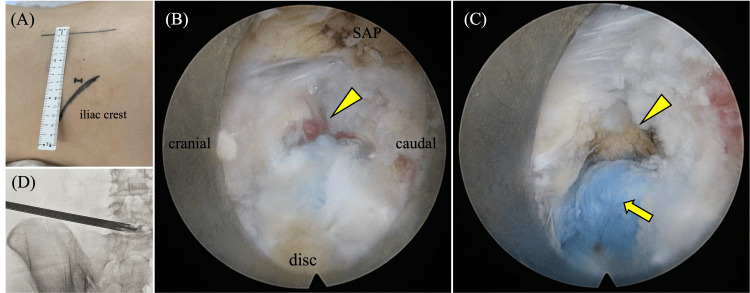
Second surgery (thermal annuloplasty). Image taken during the second surgery (thermal annuloplasty, posterolateral approach) at the L5/S1 level. (A) Entry point placed 8 cm left of the midline and slightly cranial to avoid the iliac crest. (B) Intradiscal granulation tissue with bleeding (arrowhead). (C) Scar after thermal coagulation (arrowhead) and dyed healthy intervertebral disc (arrow). (D) Intraoperative fluoroscopic image in anteroposterior view. SAP, superior articular process

The TA-PLA was performed under general anesthesia in the prone position on a radiolucent table. Motor-evoked potentials were monitored during surgery, and a marker was placed on the skin over the target disk under C-armed fluoroscopic guidance. The skin entry point was 8 cm from the midline and slightly cranial to avoid the iliac crest (Figure 5A). After sterilization and draping, thermal annuloplasty was performed according to standard procedures such as those for FED-PLA.

The entry point was infiltrated with local anesthetics, and an 8-mm incision was made. Then, the following seven steps were performed before inserting the endoscope: 1) an 18-gauge spinal needle was inserted until the posterior vertebral line under the guidance of a lateral fluoroscopic image; 2) the nucleus pulposus was stained blue by inserting a 1-mL mixture of contrast media and indigo carmine into the intradiscal space for discography; 3) a guidewire was inserted through the spinal needle; 4) the spinal needle was removed, leaving the guidewire in place; 5) a cannulated obturator was inserted along the guidewire; 6) after touching the annulus, a working sleeve with a 30-degree bevel was placed to touch the annulus along the obturator; and 7) the obturator was removed, and the endoscope (VERTEBRIS, Richard Wolf Company, Knittlingen, Germany) was inserted through the working sleeve.

Next, after making an incision in the annulus fibrosus, the endoscope was gradually advanced into the intradiscal space while being thermally coagulated using a bipolar radiofrequency probe (Trigger-Flex® bipolar system, Elliquence, Baldwin, NY). During this procedure, an “outside-in” technique was used. When the lesion showing the HIZ was reached, the presence of granulation-like tissue rich in angiogenesis was confirmed (Figure 5B). A portion of this lesion was removed using a micro-rongeur for pathological diagnosis, and the residual lesion was thermally coagulated for complete removal (Figure 5C). Lastly, a dissector was confirmed to reach the midline using fluoroscopic images in anteroposterior view (Figure 5D). The endoscope was removed after hemostasis was confirmed. Finally, the entry site was closed with a one-point subcutaneous suture and sterile dressing.

Ethical considerations

When reporting this case, care was taken to protect the patient's personal information and privacy, and consent was obtained from the patient for publication of the details contained herein.

## Discussion

A comprehensive review of the literature and prospective studies evaluating outcomes revealed that approximately 15% to 25% of patients experience recurrent LBP two years after undergoing discectomy [11]. Postoperative LBP may be associated with various factors including paraspinal muscle injuries, recurrent herniation, intervertebral disc collapse, facet joint arthritis, and injury to the posterior branch of the nerve root [12].

LBP following FED using the transforaminal approach (FED-TFA) is not rare (15-25% incidence). However, patients who underwent FED-TFA had better outcomes than those who underwent fenestration discectomy; the reported pain score after FED-TFA (VAS 1.6) for LBP was lower than that after fenestration discectomy (VAS 2.1) [13]. Nevertheless, Chen et al. reported that 8.1% of patients continued to experience persistent LBP at the two-year follow-up [14]. Thus, it is necessary to prevent or improve the management of postoperative LBP.

In several recent articles, Modic changes, injuries of the posterior longitudinal ligament, endplate abnormalities, muscle fatty infiltration, disc height loss, and intraoperative cannula positioning have been identified as significant risk factors for LBP after FED-TFA [12,15,16]. McGirt et al. indicated that overly aggressive normal disc removal is associated with higher rates of recurrent back and leg pain [17]. However, limited discectomy tends to result in a greater incidence of recurrent herniation in the long term. Wang et al. reported that the average long-term VAS score for LBP decreased to 1.08 when using intradiscal irrigation [12]. Disc irrigation helps prevent excessive normal disc removal, leading to the restoration of disc height. Both normal disc preservation and disc height restoration have shown significant associations with postoperative LBP.

The latest findings, such as histopathological (HP) changes in intervertebral discs and their association with clinical outcomes following surgical treatment for LDH, were reported in a prospective observational study by Bečulić et al. [18]. HP analysis using a semi-quantitative HP degeneration score (HDS) indicated chondrocyte proliferation, tissue tears and clefts, granular changes, and mucous degeneration. Consequently, factors such as male sex, age under 44 years, diabetes mellitus, hypertension, depression, and spinal lumbar stenosis were particularly associated with higher HDS scores. HDS was a potential predictor of postoperative outcomes for LDH, with scores ≥ 7 (median HDS of 6) indicating significant diagnostic and prognostic value for disability and LBP persistence at six months. Furthermore, HP analysis was introduced as a valuable adjunctive tool for understanding the complexities of LDH. Although the concept of HIZ was not mentioned in this latest study, we hope that a broader perspective on postoperative DLBP will lead to a deeper understanding of these issues.

As mentioned above, although there have been reports of investigations into its causes, prevention, and evaluation, there has been little mention of treatment methods after the onset of postoperative LBP, and no reports have been published on surgical treatment. In this study, we performed thermal annuloplasty for HIZ after FED. During the first surgery (FED-ILA), bleeding granulation tissue was observed inside the herniated disc. During the second surgery (thermal annuloplasty), similar granulation tissue was confirmed to be present inside the disc. The histopathological findings were largely similar to those reported previously. Previous literature on HIZ has also reported the presence of granulation tissue rich in angiogenesis; immunostaining positivity such as for CD34, TNF-α, and CD68; and the inclusion of fibroblast-like cells [19-20]. Histopathologically, HIZ is suggestive of inflammation. Hence, cauterization of the HIZ can suppress inflammation and improve DLBP. In addition, the LBP experienced before the second surgery was more severe than that before the first surgery. Nevertheless, in the histopathological analysis, there was no difference between the spontaneous HIZ in the LDH and HIZ groups after surgery.

Although the incidence of postoperative HIZ has not yet been evaluated, it appears that DLBP accompanied by an HIZ may develop after FED-TFA, and thermal annuloplasty is as effective as usual for HIZ treatment. Another advantage is that FESS is minimally invasive, making it easy for patients to return to society even after additional surgeries. This approach may be applicable not only after FED but also after fenestration discectomy.

LBP is recognized as a significant health and socioeconomic challenge worldwide, with an expected increase in prevalence. However, there is still significant potential for improvement in both its diagnosis and treatment [1]. There was a statistically significant correlation between a positive HIZ and abnormal disc morphology in discography in previous meta-analyses. Although most papers reported a high specificity for this relationship, it still remains controversial. The multiple and complex causes of LBP and DLBP results in diagnosis, management, and treatment selection difficulties. Most treatments target a single cause, and given the complex nature of LBP, diverse and multidisciplinary approaches are required. Although thermal annuloplasty for HIZ in this study may be merely one of the surgical procedures used to manage LBP originating from a single cause, it is significant since an increase in the number of useful treatment options is needed.

To our knowledge, this is the first case study reporting the use of thermal annuloplasty for postoperative HIZ. In the future, it will be necessary to increase the number of cases, observe long-term progression, and evaluate recurrence. Furthermore, future research on LBP diagnosis should focus on improving the accuracy and objectivity of diagnostic assessments.

## Conclusions

We defined “postoperative HIZ” as follows: 1) LDH that disappeared on postoperative imaging with persisting LBP, 2) no obvious findings of nerve compression and recurrent LDH ruled out, 3) MRI showing signs of HIZ, with disc block being clearly effective, and 4) any other causes of LBP not being identified. However, because distinguishing between true postoperative HIZ and postoperative scars is difficult using MRI, all of the above criteria must be simultaneously met.

Our case demonstrates the effectiveness and safety of thermal annuloplasty for DLBP with an HIZ after FED-TFA. We should conduct further research on this application of thermal annuloplasty, provide preventive measures, and determine the best therapeutic methods for postoperative LBP to obtain better treatment outcomes. We hope that this report will be of some help to patients with LBP of unknown etiology and postoperative LBP who have abandoned treatment due to unsatisfactory outcomes of conservative treatment.
